# Popular Psychological Literature

**Published:** 1857-07-01

**Authors:** 


### Art. VII.—POPULAR PSYCHOLOGICAL LITERATURE.

Amongst the various causes which have given so immense an impulse to the study of psychology, both normal and morbid, during the last few years, there is none which ranks higher, whether as an evidence or a cause of progress, than the ever increasing interchange of thought and observation between the two great classes of students who view mental phenomena from its two aspects of health and disease. The investigator of the laws of thought now sees clearly that his labour is lightened, and his results widened in extent and importance, by accepting as elements in his calculations data derived from the morbid or modified tendencies of mind; even as the true nature of many vegetable and animal developments is only to be recognised by a consideration of their extreme or *monstrous* forms. In sensual, perceptive, or emotional exaggerations, he is able to see the essential bearings of facts, otherwise probably overlooked, or neglected as of small importance. On the other hand, the mental *pathologist*,—he who contemplates mind chiefly through its disordered functions, must acknowledge, that a knowledge of the normal, is an essential element towards the correct appreciation of the abnormal; he thereby computes the nature and amount of aberration; and what is perhaps of more importance, he can calculate how much healthy *mind-material* is left in any given case; and as a corollary, he may approximately conjecture what is the probability that this healthy remainder will act favourably and therapeutically upon the disturbed functions—and thus enhance the prospect of success in the application of the "moral treatment" of insanity.

The great and important questions connected with morbid psychology, are no longer now confined to the profession; nor are those who devote themselves to their elucidation in theory and practice, looked upon with suspicion by the public; each recognises the paramount interest attached to the elucidation of such inquiries as—What is insanity? What is responsibility? What are the boundaries between physiological and pathological mental manifestations? What are the distinctions between eccentricity and aberration? What is the rational treatment for intellectual disorder? Truly important and difficult are such questions; it may be, that we are still far from being able to give satisfactory replies; that between reason and unreason, there is an extensive neutral ground, unclassified, unannexed; that many points of treatment are still obscure or *sub judice;* that an entirely new canon of responsibility is required, differing almost altogether from that now applied, all this may be; yet progress is made, and in one very important respect, viz., that the sympathy and attention of the public is gained; and instead of active opposition or passive resistance, there is now evinced a readiness to assist; or if that be impossible, to give an earnest thoughtful attention to such considerations as may be suggested. A certain sign of this growing interest, is to be found in the frequency with which allied subjects are selected for discussion in the leading periodicals of the day. It is curious to see how, without the publication of any special recent works of general interest, or the excitement attached to any remarkable case or question before the public mind, a very great proportion of the journals of the April quarter have able articles upon some subject very nearly connected with this inquiry. Thinking it of importance, from time to time, to notice the popular estimate of such matters, we propose to analyse briefly two or three of the principal papers of late appearance, with as copious extracts as our limits will allow. The able writer of the article on Lunatic Asylums, in the April number of the *Quarterly Review*, dwells at great length upon the substitution of *moral* treatment for that of *physical force* amongst the insane, contrasting the state of matters when Horace Walpole spoke in one breath of the "lions of the Tower" and the "madmen out of Bedlam," with the orderly, cheerful, almost happy communities, which now in many places represent the asylums for the insane. Some of the introductory observations are very important, as bearing upon the necessity for the *early treatment* of mental disorder; considerations, the ignorance or neglect of which, lead to such serious statistical results as to cure.

Yet in spite of the ameliorations in the condition of the insane, many among the higher, and nearly all among the lower classes, still look upon the county asylum as the Bluebeard's cupboard of the neighbourhood. These unfounded ideas act as a *powerful drawback to the successful treatment of insanity;* for as the vast majority of cures are effected within three months of the original attack, whatever deters the friends of the patient from bringing him under regimen at the earliest possible moment, *probably ensures the perpetuation of the disease.* We can well imagine the undefined awe and tribulation of spirit with which the unhappy creatures who are stricken in mind, enter the gates of an abode in which they are supposed to be given over to a durance worse than death; but so mistaken is the impression, that the feelings of desperation are *almost immediately* succeeded by the inspiriting dawnings of hope.

"The furious maniac who arrives at Colney Hatch or Hanwell in a cart, or a hand-barrow, bound with ropes like a frantic animal, the terror of his friends and himself, is no sooner within the building which imagination invests with such terrors, than half his miseries cease. The ropes cut, he stands up once more free from restraint, kind words are spoken to him, he is soothed by a bath, and if still violent, the padded room, which offers no aggravating mechanical or personal resistance, calms his fury, and sleep, which has so long been a stranger to him, visits him the first night which he spends in the dreaded asylum. . . . . . . In the old plan, the entire treatment seemed to consist in secluding the patient from every sight which renders life sweet, and in wrenching him violently from all the conditions which formerly surrounded him; the new idea is to bring within the walls as much of the outside world as possible. Here the artisan finds employment in various handicrafts, the agricultural labourer renews his commerce with the soil, and the female plies her needle, or pursues her accustomed occupations in the laundry or the kitchen. Amusement takes its turn, and those who travel by the Great Western train on winter evenings, are surprised to see the lights streaming from the great hall of Hanwell, and to hear perchance the sounds of music. These issue from the *ball-room* of the establishment."

We can scarcely attach too great an importance to the promulgation of facts and opinions like these, through the medium of so influential an organ. Nothing can be better calculated to sweep away all remaining prejudices from the mind of the public, in reference to the isolation of the insane in such establishments. The balls which have been alluded to are a very modern feature, and a very interesting one in the treatment of mental affections. They are part of one great plan of occupation and amusement; and distracting the mind from dwelling upon itself, and its own sorrows. Colney Hatch every Monday night presents a singular spectacle. About 200 of the patients are assembled in the assembly-room—males and females together—some of them are musicians, and play for the company—some dance, and some play whist—in all this there is no disorder, nor anything which would indicate that the company were lunatics; the uniform of grey alone lends an air of incongruity to the otherwise lively scene.

"Amongst the merriest dancers in Sir Roger de Coverley was a man who believed himself to be our Saviour, and who wore in his hair a spike, in imitation of the crown of thorns; and one of the keenest whist players was an old lady, who, whilst her partner was dealing, privately assured us that she had been dead these three years; and desired as a favour that we would use our influence with the surgeon to persuade him to cut off her head."*

These balls are attended with the great advantage of allowing to some extent the association of the sexes; for no life can approximate to normal sociality where the isolation is preserved inviolate. In this particular we are much in advance of our con-

* Quarterly Review, p. 575.

tinental brethren ; for although balls and other amusements are practised very extensively, the sexes are not allowed to mix at all. Such at least is the case at Stephansfeld, a large and excellent institution some leagues from the Rhine, near Bramath, an interesting account of which is given by M. Paul Janot, in the *Revue des Deux Mondes* for April 15th. Here, the balls are for the women alone, "the mixture of the sexes being rigorously forbidden." M. Janot remarks upon the general aspect of this ball room :—

"We sometimes hear exaggerated the appearance of reason which the insane present. I have even read somewhere the account of a ball at which the author was present, and passed half the night without knowing that he was amongst the insane. Certainly this mistake would have been difficult in the modest ball at which I was present. There is no disorder—the result of good discipline; but certain signs sufficiently indicate that the society is not *sane.* It is not disorder—it is sadness; it is not extravagance—it is silence. The contraction of the countenance, a certain *désaccord* in the dress, the monotony of the movements, many exterior signs betray the disorder of thought, and scarcely permit a mistake to be made."

The Bethlehem Hospital is thus noticed in the *Quarterly Review:*—

"No cases of more than twelve months' standing are admitted within the walls of Bedlam, and only 90 persons termed incurables are allowed to remain beyond that period. These regulations exclude the idiotic and epileptic patients, who form such distressing groups in other establishments, and the interest required to obtain admission into this amply endowed charity, insures at the same time a much higher class of inmates. Clergymen, barristers, governesses, literary men, artists, and military and naval officers, make up the staple of the assembly. There is a ball on the first Monday in every month, and the company that gathers in the crystal chamber, at the extreme end of the south wing, would not disgrace in behaviour and appearance any sane and well-bred community. The polka, the waltz, and the mazurka, performed with grace and ease, declare the social standing of the assembly; and many a pedestrian who sees the dark silhouettes of the dancers, as they whirl across the light, is astonished at the festivities of the inmates. In the summer evenings, the spacious courts are crowded with the patients, not gloomily walking between four dismal walls, in which the very air seems under restraint, but enjoying themselves in the bowling-green or in the skittle-alley. When we contrast the condition of the Bethlehem of fifty years ago with the Bethlehem of to-day, we see at a glance what a gulf has been leaped in half a century—a gulf on one side of which we see man like a demon torturing his unfortunate fellows; on the other like a ministering angel carrying out the all-powerful law of love."

Music, painting, reading, conversation for the men; every variety of needlework for the women, "dividing their attention with the young lady who reads aloud 'David Copperfield' or 'Dred;'" such are the occupations of the patients at Bethlehem. At Colney Hatch and Hanwell, refinement in treatment is not carried to the same extent; there is the same kindness and rationality; but whilst in the former, a quarter of a million sterling has been expended principally upon the outside of the building, "not a sixpence can be spared to adorn the walls within, with either picture, bust, or even the commonest cottage decoration. This is the vice which pervades the majority of county asylums lately erected."

The question of restraint is very temperately discussed in the article from which we have quoted so largely. The writer cannot "from a fancied apprehension of the return to obsolete practices join in the fanaticism which forbids the use of the strait-jacket, as a means of coercion under all circumstances. There can be no doubt that the treatment which requires its frequent use is a bad one; but to deny that there are cases which call for its restraints would be to deny the evidence of our senses."

*What is to be done with criminal lunatics?* A very difficult problem is this to solve. At present their dens appear to be the only remains of the old system of treatment. Their dens consist of "dismal, arched corridors, feebly lit at either end by a single window in double irons, and divided in the middle by gratings more like those which enclose the fiercer carnivora at the Zoological Gardens, than anything we have elsewhere seen employed for the detention of afflicted humanity."

"Here fifty male lunatics are herded together, without regard to their previous social or moral condition. Thus the unfortunate clergyman, the Rev. Hugh Willoughby, who fired a pistol two years since at the judge of the Central Criminal Court, is herded with the plebeian perpetrator of some horrible murder. Side by side with the unfortunate Captain Johnson of the ship *Tory*, who in a fit of extraordinary excitement during a mutiny on board his vessel, cut down some of his crew, but is now perfectly sane, sits perhaps the ruffian who murdered the warder in cold blood at Coldbath-fields—a villain brought in mad by a tender-hearted jury, who shrunk from the responsibility of hanging him. Here also poor Dad, the artist, who killed his father whilst labouring under a sudden paroxysm of insanity, is obliged to weave his fine fancies on the canvas amidst the most revolting conversation and the most brutal behaviour. The disgrace of thus caging-up together the coarse and the gentle, the virtuous and the abandoned, rests wholly upon the shoulders of the Home Secretary. It is proposed to build a special asylum for all the state lunatics; steps are being taken, we believe, to effect this necessary change; but unless Parliament puts its pressure upon the Home-office, we shall expect to see the arrangement completed when the Nelson column is finished, and not before."

On the subject of the town or country location for hospitals devoted to the cure of mental affections, there are more observations, which, though correct enough, do not appear to exhaust all the considerations that ought to have weight in the decision.

The writer* opposes the removal of Bethlehem Hospital into the country, on many grounds—that agricultural pursuits are neither necessary nor desirable for the class of patients contained there—that the sights and sounds of the metropolis, which they are now enabled to enjoy, afford them more recreation than wading through ploughed fields, or taking a turn at spade husbandry—that mental affections are often sudden seizures, and require prompt aid, as well as the frightful casualties of the streets requiring surgical aid.

"It would not, perhaps, be undesirable to add to Bethlehem some small rural establishment, answering to the *succursales* of foreign lunatic asylums; but this should be strictly an appendage, to which patients should be sent for a short time, for change of air and scene, just as all the world now and then take a trip to the country to refresh the wearied eye with the sight of green trees and fields, and to cure that moral scurvy contracted by perpetually dwelling upon the dismal vistas of blackened bricks which constitute metropolitan prospects."

It cannot be doubted that there are certain advantages derivable from a town location for hospitals of all kinds, not excluding even those devoted to mental affections; but against these considerations, it must be remembered that the conditions of life and health are much more unfavourable in city districts than in those of the open country. As deduced from the reports of the Registrar General, the mortality of equal populations in towns and country districts, is about in the proportion of 2 or 2½ in the former, to 1 in the latter; if we take the metropolis, and compare it with some of the districts of the southern counties, the disproportion is still more striking. On further analysing the statistics, it may clearly be demonstrated that affections of the nervous system afford even more than their full or legitimate quota to the production of this excessive mortality in towns. It needs, apparently, then, no argument to prove, that as mental alienation is but one form, perhaps the culminating form in which the effect of degenerating influences is manifested—and as these influences are shown to be especially rife in cities—so the prospect of being able to counteract these effects will (*cæteris paribus*) be in proportion greater, as the individual affected can be removed from the sphere of their operation. Doubtless many other elements must enter into the solution of so extensive a question; but of these, such statistical considerations as the preceding must by no means be esteemed the least urgent. M. Paul Janot speaks very decidedly on this point:—

* Quarterly Review, p. 362.

"Nothing is insignificant in the construction or constitution of an asylum for the insane. Everything ought to be prepared to remove false associations, to suggest true ones, to soothe and annul painful and irritating impressions, and to favour the development of pleasant and serene emotions. In this point of view, one of the first and most essential conditions is *a situation in the country, and in good free air*. This is one of the advantages of the house of Stephansfeld; it is surrounded by fields and forests; enclosed in a garden, the limits of which are ingeniously concealed. The view is vast and beautiful; there are no mighty and sombre mountain aspects, such as strike the imagination of the artist or the poet; but which would be very imperfectly suited to the disordered mind; but everywhere nature is smiling but *ordinary*. What is especially salutary, however, is not so much the beauty of the site, to which the eye soon becomes habituated, but the insensible influence of an open and pure atmosphere."

After dwelling at considerable length upon the internal economy of the public asylums, in a manner eminently calculated to remove from the mind of general readers any ideas prejudicial to such institutions; the writer in the Quarterly proceeds to notice the condition of private establishments for the insane.

"The licensed houses in the metropolitan district directly under the control of the Lunacy Commissioners, amounting to forty-one in number, represent without doubt the fairest specimens of these establishments. Liable as they are at any moment to the inspection of the Commissioners, and presided over, as many of them are by the most eminent members of the profession, they are generally maintained in a high state of efficiency. They are principally devoted to the cure of the higher classes of the community, and afford, perhaps, the nearest approach yet made to a perfect method of treatment, being conducted in most cases on the principle of a private family. The bolts, bars, high walls, and dismal airing-courts of the public asylum are either unknown, or so hidden as no longer to irritate the susceptible mind of the lunatic. The unwise division of the sexes is rarely adopted. Scrupulous attention to dress and all the forms of polite society are enjoined alike for their own sake, and as a method of interesting the patients in the daily life of the community. When we partook of the hospitalities of one of these establishments, we could detect nothing in the countenances or the appearances of the guests which was characteristic of their condition—the restless eye, the incoherent conversation, the sudden movement of the peculiarly formed head, which our preconceived notions led us to expect, were none of them observable. One individual there was, indeed, whom we mentally concluded to be certainly mad. Yet, singular to say, this gentleman was the only sane individual in the room besides ourselves and the medical superintendent; and on further acquaintance, having told our ill-placed suspicions, he frankly confessed that he had in his own mind paid ourselves a similar compliment. The eager glance of curiosity natural to inquisitive strangers, was the nearest approach in this lunatic party to the outward appearance of lunacy. So much for the 'unmistakeable countenance' of the insane! It is not to be supposed that the more violent can be allowed this social freedom even in private establishments, or that madness is different in a metropolitan licensed house from what it is in a public asylum; but we unhesitatingly assert that, in the vast majority of cases, the large amount of freedom, and the absence of any prison-like characteristics have an undoubted effect, not only in calming the mind of the patient, but in expediting his recovery. Hence the per centage of cures in a high-class private asylum are immeasurably beyond those of any public establishment. The pleasure ground, out-of-door games, carriage and riding parties, billiards, whist, and evening parties, all contribute their aid in restoring the unhinged mind. We have seen four or five patients leave the doors of one of these licensed metropolitan houses, and remain out for hours, without any attendant; their word of *honour* being the only tie existing between them and the asylum."

With reference to coercion or restraint, it appears that we are far in advance of our continental brethren; a statement fully proved by reference to Dr. Webster's statistics recently published in this journal. The writer adds:—

"When the beneficent thought struck the great Pinel to knock off the fetters of the English captain, he sounded a note which reverberated through Europe, and the poor insane captives issued from their dungeons in which they had been so long immured, as the prisoners emerge from their prison to the divine strains of Beethoven's 'Fidelio.' But when this vast step was accomplished there still remained an immense amount of coercion scarcely less injurious than the old darkness and chains, and to Englishmen is mainly due the credit of abolishing it. Nor shall we rest where we are. It is our belief, as well as our hope, that before another generation has gone by, the last vestige of restraint, in the shape of dismal airing-courts, and outside walls, which serve to wound the spirit rather than to enslave the limbs, will pass for ever from among us, and only be remembered with the hobbles and manacles of the past."

The last subject treated upon in this able paper refers to the alleged increase of lunacy:—

"It has been asserted by some psychologists that lunacy is on the increase, and that its rapid development of late years has been consequent upon the increased activity of the national mind. This statement is certainly startling, and calculated to arrest the attention of all thoughtful men. Is it true that civilisation has called to life a monster such as that which appalled Frankenstein? Is it a necessity of progress that it shall ever be accompanied by that fearful black rider which, like despair, sits behind it? Does mental development mean increased mental decay? If these questions were truly answered in the affirmative, we might indeed sigh for the golden time when

'Wild in woods the noble savage ran,'

for it would be clear that the nearer humanity strove to attain towards divine perfection, the more it was retrograding towards a state inferior to that of the brute creation. A patient examination, however, of the question entirely negatives such a conclusion."

The line of argument adopted is, that were such the case, the principal sufferers should be found in those classes of society which are in the van of civilization—amongst bankers, great speculators, merchants, engineers, statesmen, philosophers, and men of letters—those who work with their brain rather than hands. Such increase then would naturally be sought for in *private* asylums, which are especially adapted to the upper classes of patients; yet between 1847 and 1855, notwithstanding the increase of population, there was a positive decrease of 96 in the entire numbers—from 4649 to 4557; whilst in the public or pauper lunatic asylums, the reports indicate an increase of 64 per cent., during the same period.

"These figures, if they mean anything, prove that it is not the intellect of the country that breeds insanity, but its ignorance, as it cannot be for one moment contended that the great movements now taking place in the world originate with the labouring classes. . . . . If we required further proof of the groundless nature of the alarm that mental anxiety was destroying the national mind, we should find it in the well-ascertained fact that the proportion of lunatics is greater among females than males. It may also be urged that Quakers, who pride themselves on the sedateness of their conduct, furnish much more than their share; but for this singular result, their system of intermarriage is doubtless much to blame. . . . . Still the fact remains that within a period of eight years, an increase of sixty-four per cent. took place in our pauper lunatic asylums. These figures, however, afford no more proof of the increase of pauper lunatics than the increase of criminal convictions since the introduction of a milder code of laws, and the appointment of the new police, afford a proof of increased crime. Medical practitioners of late years have taken a far more comprehensive as well as scientific view of insanity than formerly; and many forms of the disease now fall under their care, that were previously overlooked, when no man was considered mad unless he raved, or was an idiot. Bnt the great cause of the increase of lunatics in our asylums is to be ascribed to the erection of the asylums themselves. These establishments, in which restraint, speaking in the ordinary acceptance of the term, is unknown, and in which the inmates are always treated with humanity, have drained the land of a lunatic population which before was scattered among villages or workhouses, amounting, according to the computation of the Commissioners, to upwards of 10,500—just as the deep wells of the metropolitan brewers have drained for miles around the shallow wells of the neighbourhood in which they are situated."

There is no stronger evidence of the increasing hold gained upon the public mind by a conviction of the importance of questions connected with morbid psychology, than its commencing recognition as an essential element in forming a true judgment concerning morals, habits, and certain phases of quasi-religious development. Though the time has for ever passed away when crazy old women were burnt or drowned as witches—though it is no longer the fashion to see in every case of raving mania, demoniacal possession, even amongst the rigorous spiritualists—yet it is still very frequently the case that influences and results of a purely physical nature, as hallucinations of eye or ear, visions and audible voices are mistaken for revelations or temptations; and that depressed or exalted conditions of the nervous system which have a clearly demonstrable natural cause, are viewed as sureties of some spiritual blessing, or threats of utter spiritual ruin. Errors of this nature can but be sources of injury to true religion; and it is with especial pleasure that we notice any attempt, emanating from whatever source, to correct them—to show that states of ecstasy are no more to be viewed as marks of supernatural divine favour, than deep despondency is to be considered as a sign of reprobation; and to indicate the very important part played by the physical part of our nature when it trenches upon the psychical. The *London Quarterly Review*, a journal in close connexion with, and published under the auspices of, a very numerous and highly influential religious body, has dared boldly to advocate these principles; and in an able article, entitled "Insanity, Disease, and Religion," has ventured to assert the claims of the body to consideration, even in questions hitherto considered essentially spiritual, in a manner eminently calculated to lead to more correct views of many phases of mind, and to remove stumbling-blocks from the paths of many sincerely pious, though not very scientific people; although the writer will doubtless appear, to those who allow *reason* no share in judging of revelation, as venturing too far towards materialism. We propose to give some copious extracts from this paper, as indicative of the present state and future prospects of this particular branch of popular psychology. It must be premised that one principal object of the writer is to combat the notion that religion is a frequent cause of insanity.

"Before proceeding to illustrate supposed cases of religious insanity, we will show how the spiritual condition is influenced by disorders of the body. . . . . The comfort and efficiency of the intellect, nay, the moral perception, manliness, and virtue of the mind, depend greatly on our use of aliment; and in the very means by which we sustain the strength of the body, or most directly disorder its functions, we at the same time either fortify or disable the brain. It is of course known that the physical nature of man depends upon his food; but it is less known how much his moral nature depends upon the physical; or what changes in the temper and disposition are produced by physical influences. . . . . It has been said, and probably with truth, that food has a higher bearing on the mind than on the physical frame of man. It has been shown experimentally that the mind can only exert its powers through the instrumentality of the bodily organs. . . . . And from the doctrine deducible from such facts as these, it follows that every fresh inroad upon the mind, every example of amentia, delusion, or insanity is connected with some corresponding change in the condition of the body.* Dr. Cheyne remarks, that he never 'saw a case of mental derangement, even when traceable to a moral career, in which there was not reason to believe that bodily disease could have been detected before the earliest aberration had an opportunity offered for examination;' and the same highly religious and scientific authority adds, 'not only does every deranged state of the intellectual faculties and the natural affections depend upon bodily disease, but also derangements of the *religious and moral sentiments* originate in diseases of the body.' Hence it can be explained that the sinking of despair is not more dreadful or extreme than the hopelessness which depends merely upon the diseases of the *nervous system*. . . . . Perhaps it may startle some to be told that even *the conscience*, which is popularly supposed to be the faculty most of all independent of physical causes, is yet affected by health and disease. Facts, however, seem to place this theory beyond dispute. Examples are found in such as indulge excessively in the use of ardent spirits, opium, tobacco, and other narcotics, which become insensibly attractive, partly from habit, and partly from *loss of mental energy*, caused by their acting injuriously on the nervous system. It is also known to be matter of daily observation by persons whose profession throws them in the way of such cases, that men who were originally honourable and honest become false and dishonest through habits of intemperance, and at last have their consciences deadened, as if seared with a red hot iron. . . . . That the conscience is more or less active according to the condition of the body is illustrated by the state of the latter when exhausted by pain or sickness, or even fatigue; the conscience is then less sensitive, and in that half dreamy state that precedes sleep, especially after great fatigue, trains of thought or lines of conduct, are allowed to pass through the mind in review, which would be at once rejected were the body in vigour and the conscience on the alert. . . . .

"In proceeding to give a few sketches of insanity, in supposed connexion with religion, in the hope of aiding the inexperienced guide, it is obvious to remark, that the forms of *its approaches* chiefly require to be understood, as the confirmed disease itself lies wholly beyond his department. The following case will illustrate the value of this kind of information, which we believe would be wholly mistaken, and

* It will of course be understood that in giving these quotations, we in no way pledge ourselves to the precise scientific views adopted therein. They are given simply as an illustration of the popular tendency of the present time, and of the growing disposition to allow certain opinions due weight. The article in question is, we believe, written by a non-professional scribe—the more valuable on that account for the present purpose.

treated with erroneous measures, by one who had not been initiated in the theory we are propounding: 'Such a state as mine you are probably unacquainted with, notwithstanding all your experience. I am not conscious of the suspension or decay of any of the powers of my mind. I am as well able as ever I was to attend to my business; my family suppose me in health; yet the horrors of a madhouse are staring me in the face. I am a martyr to a species of persecution from within, which is becoming intolerable. I am urged to say the most shocking things, blasphemous and obscene words are ever on the tip of my tongue; hitherto, thank God, I have been enabled to resist; but I often think I must yield at the last, and then I shall be disgraced and ruined for ever. I solemnly assure you, that I hear a voice which seems to be within me, prompting me to utter what I should turn with disgust from if spoken by another. If I were not afraid you would smile, I should say there is no accounting for these extraordinary articulate whisperings, but by supposing that an evil spirit has obtained possession of me for the time. My state is so wretched, that compared with what I suffer, pain or sickness would appear but trifling evils.'

"A somewhat similar case occurred within our own experience, with which religion was so mixed up as to lead to a suspicion of demoniacal possession. We visited the person almost daily for many weeks, and had to listen to the same sorrowful account of her temptations to utter blasphemous words and oaths, and of her struggles to repel the most impure suggestions. The case proved to be strictly a medical one, as we told her from the first; though it gave ample opportunities for instruction and warning afterwards. . . . . The object of citing these and similar cases is to verify the medical opinion, that mental derangements are invariably connected with bodily disorder; and that the Christian teacher has but little encouragement to put Divine truth before a melancholic or hypochondriacal person, until the bodily disease with which the mental delusion is connected is removed."

We can scarcely overrate the importance of the tendency manifested by observations such as these, emanating from the very midst of a body of eminently pious and devoted men, who have hitherto manifested perhaps too great a reluctance to take any views of such questions except those which had a most strictly spiritual bearing. The remarks that follow form a just corollary.

"Hence it is clear that a case is often referred to religious despair, which in truth is to be accounted for by the absence of the controlling influences of religious principles. The Christian who is ignorant of the laws by which the human body and mind are hedged in, or careless of observing them, may easily bring on diseases which will tend to render the conscience obtuse, destroy hope, and cut short his days, or deprive him of his reason. For religion frees not its most ardent votary from the yoke of physical laws. If for the sake of subduing the flesh, or of obedience to ecclesiastical discipline, extreme fasting is practised, the penalty will be exacted at some time, as the premature death by consumption of many an enthusiastic female has proved. And just in the same manner, if the true servant of God, disregarding the laws of the body, tasks it beyond its powers, even for the noblest ends, premature decay or dissolution will be the penalty. And the literary man goes to his work under the same unalterable conditions. The brain of every man is constituted to perform a certain amount of labour only, without receiving injury; and therefore all beyond this must entail evils which it is plain from analogy, may accumulate by repetition until its ruin follows.* Abuses of the laws of the digestive organs will in the same way accumulate by repetitions, until this instrument, by which life is built up, becomes virtually destroyed, or unequal to its necessary functions."

We will close our extracts from this paper by the following remarks on those illusions or hallucinations which occasionally attend the close of life :—

"Another example of the effect of disordered functions is not uncommon to the visitant of the dying chamber. We ourselves have had to listen to it as a proof of the soul's safety in death, that during the night the sick sleeper saw beautiful sights of waters and gardens, and heard angelic melodies. The experienced physician at once confidently consigns such cases to the class of delusions to be accounted for by physical laws. Far stronger claims than the above to what after all, if they be true, must amount to a divine revelation, are confidently referred to delusions of the senses. It is certain, however, that lasting moral changes have occasionally followed such scenes (as in the remarkable case which resulted in the conversion of Col. Gardiner); and a very high authority, Jonathan Edwards, aware of the difficulty they presented to some minds, but confident of their natural origin, states his judgment thus: 'It is possible that such suggestions may be the occasional or accidental cause of gracious affections; for so may a mistake and a delusion.' This decision seems to place such cases on their true footing. We feel we are treading on dangerous ground; but the facility of the abuse of such airy nothings as dreams, which every night must produce in myriads, involving awful dangers to the immortal soul, is so great, from the natural credulity of the human mind, and from its preference for such cheap evidence to the more costly but only true evidence of real repentance, trust in Christ, and the indwelling influences of the Holy Spirit, witnessed by change of life and conversation, that we deem it needful to be able to speak with confidence and decision. In cases, however, in which a spiritual guide may feel confident that an hypothesis of demoniacal possession is wrongly assumed, and that the beautiful sights and angelic sounds are of the earth earthy, the difficulty will still remain, how to convince the poor deluded sufferer, that both the anguish and the joy are alike without a spiritual basis. In particular cases, however, this has been effectually accomplished by explaining the causes which harass the sight during

* We believe these observations have special bearing upon the melancholy end of Hugh Miller.

disease; that sparks, flashes of fire, haloes, and the like, are produced by disorders of the optic nerve or the brain; and that discordant noises or articulate sounds depend solely upon accelerated circulation through the brain or affections of the auditory nerve. By medical treatment and clear explanations of natural causes and effects, persons who supposed themselves demoniacally possessed—given over to Satan,—have been relieved from excruciating perplexities. Or, as it has been more tersely expressed, 'Cure the choler, and the choleric operations of the devil will cease.'"

The "Irish Quarterly Review" deals elaborately with "Suicide, its Motives and Mysteries," but our limits do not permit us to enter into any analysis of the paper. This is of the less consequence, as the illustrations are for the most part drawn from cases which have already been detailed in these pages.

Nor can we at present notice more fully M. Paul Janot's very interesting account of the asylum at Stephansfeld. He takes occasion to inquire into the relations between reason and insanity,—how much of the former remains to some extent unimpaired even in confirmed cases of the latter—in a manner highly philosophical, and worthy of future attention.

But we cannot close a paper in which so much has been said on the subject of the mild and benevolent treatment of the insane, without glancing at the reverse picture,—the treatment of lunatics in Scotland, as developed in the "Report of the Scottish Lunacy Commission." Our notice is extracted from the leading articles in the "Times" for May 30th and June 1st, as we could not, by any words of our own, bring the subject more fully and impressively before our readers.

"The old-fashioned treatment of lunatics, as developed in the 'Report of the Scottish Lunacy Commission,' was brought before the House of Commons last night by Mr. E. Ellice. This system is happily so obsolete in this country that we rank it with the barbarisms of the middle ages. Handcuffs, leg-locks, gloves, straps, and strait-waistcoats are as antiquated weapons to use in the warfare against insanity as bows and arrows are in common war. But, according to this Report, and according to Mr. E. Ellice, this is still the main system in use in Scotland. It is indeed surprising to see how completely a mere arbitrary boundary line stops the advance of an important improvement; and yet Scotland, with its great medical school, is the last country in the world where we should have expected such barbarisms to be maintained; for the new treatment of the insane, though a benevolent movement in some degree, is mainly a medical discovery. Scientific men discovered that the old system was a mistake—that madness was not to be met by such remedies; and the new system grew up as any other medical improvement might—such as the new mode of treating fever. As the old established seat, then, of medical science, why did not Scotland take the lead in this discovery, instead of being, as the fact turns out, the very last even to take advantage of it when it has been made?

"The institutions called 'chartered asylums' in Scotland seem tolerably free from this charge. The Commissioners, indeed, object to the 'cage-like' appearance of these structures, which are 'enclosed externally by strong wire or light ironwork.' They object to their 'long galleries radiating from the central staircase,' and recommend 'more simple and ordinary buildings for the poor, having a more domestic aspect and arrangement.' The very sensible language of the Report, indeed, on this point, deserves to be quoted:—

"'There is little doubt that to be near home, and to be surrounded with homely objects, in dwellings having a domestic character, and affording opportunities for ordinary daily occupation in household work, by arrangements familiar to them at home, are grateful to the feelings of poor patients, who generally prefer an inferior description of accommodation of this kind to the spacious galleries provided in some of the public asylums. In such plain domestic buildings a more contented frame of mind is likely to arise. These apparently trifling arrangements assume a degree of importance when it is considered that by recalling past impressions, awakening deadened sympathies, and reviving former habits and customs, they may become the means of arresting the aberration of a diseased mind and of restoring it to healthy action.'

"These chartered asylums use the expedient of seclusion too much; but they appear to have dropped the coarser weapons of the old system. The 'licensed houses,' however, retain the old coarse system of instrumental restraint. These are establishments set up by persons as private speculations, and often, as the Commission complains, by unfit persons, the Sheriff not seldom giving licences to men who have no professional knowledge of the subject. The keeper of one of these establishments at Musselburgh had been a 'victual dealer,' another had been an 'unsuccessful baker,' another had been a 'gardener,' another 'a woman who had kept a public-house.' Instrumental restraint is in very general use in these houses, and is applied to private patients as well as pauper ones. 'There are houses in which some of the paupers are kept constantly manacled.' The straitwaistcoat is in daily use. 'In almost every house we found,' say the Commissioners, 'handcuffs, leg-locks, gloves, straps, and straitwaistcoats, and these not in the custody of the proprietor or medical attendant, but hanging up in the wards.' The Commissioners discovered that the patients were 'restrained by means of manacles, fastening the arms behind the back, and also to rings fixed in the wall.' The seclusion-room comes in as supplementary to this system. 'In the Barony workhouse a very narrow slit admitted light and fresh air into three rooms, which were thus close, dark, and offensive.' The patient in seclusion lies on a mattress on the floor, or on loose straw covered by a sheet.

"The sole motive, of course, in setting up these establishments is profit, and 'the accommodation of the greatest possible number at the smallest outlay' is the great aim. There is consequently overcrowding, with an absence of proper separation of male and female patients.

'Most of the pauper houses have no day-rooms, the patients when not in the airing-grounds occupying their crowded sleeping-rooms during the day,' and some which have day-rooms hardly furnish them. In very few are there any single rooms for the separation of the epileptic, noisy, or refractory. Even the upper class of patients are often miserably lodged:—

" 'Two male patients were confined in Hill-end Asylum, near Greenock; both had occupied respectable positions in life, and the payments made for them were respectively 53*l*. and 35*l*. per annum. At the time of our visit they shared a small bedroom with a third patient, and for months had slept together, entirely naked, in a miserable trough bed, upon a quantity of loose straw.'

" The dormitories of these over-crowded establishments are, of course, abominable; and whereas in Lanarkshire the Sheriff had fixed 800 cubic feet of air as the *minimum* allowance for each patient, the allowance in some cases is only 200, and the average is only 300—and this with hardly ever any arrangement for ventilation other than the natural outlets of the room afford, and even these not used. 'The windows, even in summer, are almost always closed during the night, and the fireplaces are generally boarded up, so that ventilation is impossible.' Very few of these establishments possess a warm bath, and even the ordinary washing accommodation is exceedingly defective. 'Frequently there are no basins, and the patients wash in tubs, or at the pump; but in some cases it seemed doubtful whether they washed above once or twice a week.' In Lilybank this tub was placed 'in a damp shed, which served also for the deadhouse.' The table linen was often extremely dirty, and in some houses the patients 'were served in their sleeping-rooms, taking their food in a basin, and tearing it with their fingers.' These establishments also are grievously deficient in space, and do not give room for necessary exercise and amusement. Everybody knows what important ingredients these are in the good treatment of insanity, and that the want of them is fatal. Yet the grounds for 60, 70, 80, or 90 patients rarely exceed one acre in extent, while there is seldom any attempt made to provide the men with any kind of work or amusement.

" Such is the picture which Mr. E. Ellice, relying upon this Report, gives of the present treatment of lunatics in Scotland—a country which, though blest with two Church Establishments, a body of stipendiary Sheriffs, and a judicial Bench quite out of proportion to the work it has to discharge, seems to have known nothing of these abominations. The disclosures of this Report will be received with surprise and indignation by the English public, which has been now so long accustomed to a better system, and which will, we trust, insist on immediate legislation to remove a practice which is a stain on the character of Scotland and a disgrace to our common humanity."

June 1st:—

" The remarks which we have made apply in some degree to all treatment of the insane in Scotland, those of the better as well as those of the lowest classes. The Scotch public has not yet caught the true aspect in which insanity should be regarded, and, this being the case no system of supervision and inspection could ever substantially improve their treatment of the insane. Any Board of Supervision would be stopped by the thickness of the material they had to deal with and the passive resistance of ignorance and rudeness. The Board of Supervision at Edinburgh of course depends upon the reports of agents in the districts, upon subordinate officials on the spot, Poor Law officers, and parochial Boards. But these local authorities are under the same vulgar influences which affect the class they have to control. They see no particular harm in these cases, and neither report them to the Board nor take any steps to correct them themselves. Thus the whole work is left to the natural temper and ideas of the people.

The only arrangement which can work any real reform in the treatment of the insane in Scotland is the establishment of county and borough lunatic asylums there, to be supported by rates, as in this country. This will not only furnish places under regular scientific management for the reception of the insane, but it will do what is the great thing wanted—give the Scotch a new idea about insanity and its treatment, and thus raise the standard of treatment even in private asylums. The existing chartered asylums are too few to have this general effect."

These statements carry with them their own comment. The facts to which they relate are only able to exist in darkness—their revelation will, we feel assured, be speedily followed by their amendment.

---

